# Involved‐field high‐dose chemoradiotherapy with respiratory motion management for esophageal squamous cell carcinoma

**DOI:** 10.1111/1759-7714.15468

**Published:** 2024-10-11

**Authors:** Masaki Matsuda, Takafumi Komiyama, Kan Marino, Shinichi Aoki, Tomoko Akita, Naoki Sano, Hidekazu Suzuki, Masahide Saito, Hikaru Nemoto, Hiroshi Onishi

**Affiliations:** ^1^ Department of Radiology University of Yamanashi Chūō Yamanashi Japan

**Keywords:** dose escalation, esophageal carcinoma, esophageal fistula, involved‐field radiotherapy, respiratory motion management

## Abstract

**Background:**

We investigated the clinical outcomes of involved‐field high‐dose (≥66 Gy) chemoradiotherapy (CRT) combined with respiratory motion management for esophageal squamous cell carcinoma (ESCC).

**Methods:**

Patients who underwent definitive CRT for histologically confirmed ESCC in our department between 2012 and 2018 were retrospectively analyzed. Respiratory motion management strategies included breath‐holding (63%) and mask immobilization (29%) based on individual measurements of respiratory tumor motion using radiographic fluoroscopy with endoscopically placed clip markers as landmarks. We evaluated patient characteristics, treatment efficacy, failure patterns, and toxicities.

**Results:**

We enrolled 35 patients with a prescribed dose of 66–70 Gy in 33–35 fractions. The overall response rate within 6 months post‐CRT was 94.3%; the median follow‐up period for survivors was 43 months. The 2‐year overall survival (OS), progression‐free survival, and locoregional failure‐free survival rates were 51.4%, 42.9%, and 42.9%, respectively. A significant difference in OS was observed between patients with and without esophageal fistulas after CRT (*p* = 0.002, log‐rank test). Disease failure occurred in 16 patients (45.7%), including one (2.9%) with out‐of‐field regional nodal failure. Major grade 3 or higher toxicities included decreased white blood cell count (48.6%), neutrophil count (34.3%), and esophageal stenosis (31.4%). No grade 3 or higher cardiopulmonary toxicities were observed. Bronchial/tracheal tumor compression and a higher radiotherapy dose (70 Gy) were significantly correlated with esophageal fistulas.

**Conclusion:**

Involved‐field high‐dose CRT with respiratory motion management may be a feasible treatment option for ESCC. However, a comprehensive assessment of esophageal fistula risk is required to identify suitable candidates.

## Graphical abstract

Involved‐field high‐dose (≥ 66 Gy) chemoradiotherapy with respiratory motion management for esophageal squamous cell carcinoma resulted in a 2‐year overall survival rate of 51.4% and a low total failure rate of 45.7%, with only 2.9% exhibiting out‐of‐field regional nodal failure. However, esophageal fistulas, associated with bronchial/tracheal tumor compression and higher doses (70 Gy), significantly worsened survival outcomes. Evaluating fistula risk is crucial for dose escalation feasibility.

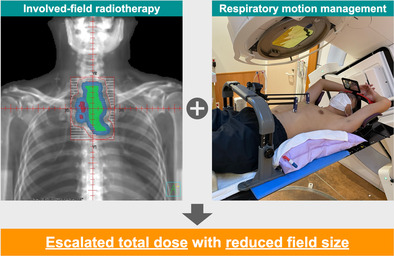

## INTRODUCTION

Esophageal carcinoma (EC) is the seventh most prevalent cancer worldwide and has a poor prognosis.^1^ In Japan, an estimated 25 920 individuals were newly diagnosed with EC in 2018, and 10 981 died from this disease in 2020.^2^ Among these patients, squamous cell carcinoma (SCC) is the predominant histological subtype of EC, accounting for approximately 90% of all cases. Esophagectomy is highly invasive and likely to reduce the quality of life; therefore, radiotherapy (RT) has emerged as a less invasive strategy, especially with the aging population.

Definitive chemoradiotherapy (CRT) is the standard treatment for unresectable EC; it offers an alternative radical treatment for patients who decline surgery or are medically unfit.^3^ The Radiation Therapy Oncology Group (RTOG) 85‐01 trial showed a statistically significant survival benefit of CRT over RT alone (5‐year overall survival [OS]: 26% vs. 0%, respectively).^4^ Furthermore, RTOG 94‐05 compared the OS between radiation doses of 64.8 and 50.4 Gy. A higher radiation dose did not improve OS, and the standard radiation dose for definitive CRT was 50.4 Gy.^5^ However, the optimal radiation field and dose remain unclear due to the high rates of local failure and distant metastasis. Recent studies have explored the efficacy of dose‐escalation beyond 60 Gy with involved‐field radiotherapy (IFRT) by omitting elective nodal irradiation (ENI)^6^, ^7^, ^8^; however, little evidence is available regarding doses of ≥66 Gy.

In our institution, we perform IFRT combined with respiratory motion management, primarily using the breath‐hold technique, to minimize the radiation field and enhance total dose delivery to the targets for improved local control without increasing toxicities. Therefore, in this study, we aimed to evaluate the clinical outcomes of patients with esophageal squamous cell carcinoma (ESCC) definitively treated with high‐dose (≥66 Gy) IFRT combined with respiratory motion management.

## METHODS

### Study design

This study was conducted in accordance with the ethical principles of the Declaration of Helsinki and was approved by the Institutional Review Board of the University of Yamanashi (approval number: 2750).

We included 45 consecutive patients who underwent definitive CRT for histologically confirmed ESCC in our department between 2012 and 2018. Patients were excluded if they had other uncontrolled malignant tumors (*n* = 6) or received a planned radiation dose of <66 Gy (*n* = 4). Ultimately, 35 patients were included in the analysis. Prior to treatment, all patients underwent assessment and staging according to the eighth edition of the Union for International Cancer Control (UICC) TNM classification. Diagnostic procedures included upper gastrointestinal endoscopy and computed tomography (CT), with bronchoscopy performed when necessary to assess bronchial/tracheal invasion. Although cervical and thoracic EC are often discussed separately owing to differences in surgical indications and approaches, this study focused on evaluating definitive CRT; therefore, both types were analyzed.

### Radiotherapy

Treatment planning was performed using three‐dimensional conformal radiotherapy (3D‐CRT) or intensity‐modulated radiation therapy (IMRT) with volumetric‐modulated arc therapy. All patients were treated with conventional fractionation (2 Gy per fraction; one fraction daily, and five fractions weekly) with a total dose of 66–70 Gy in 33–35 fractions according to the physician's judgment, with the point prescription for 3D‐CRT and the volume prescription for IMRT. Of the five patients who underwent IMRT planning, the prescribed isodose line was required to cover >95% of the clinical target volume (CTV) in two patients and 50% of the planning target volume (PTV) in three patients. Planning CT scans were acquired using GE HiSpeed DX/i (2012) and Toshiba Aquilion LB (2013–2018) with a slice thickness of 2 mm. Treatments were delivered using a 6–10‐MV linear accelerator (Elekta Synergy [Elekta AB, Stockholm, Sweden]).

**FIGURE 1:**
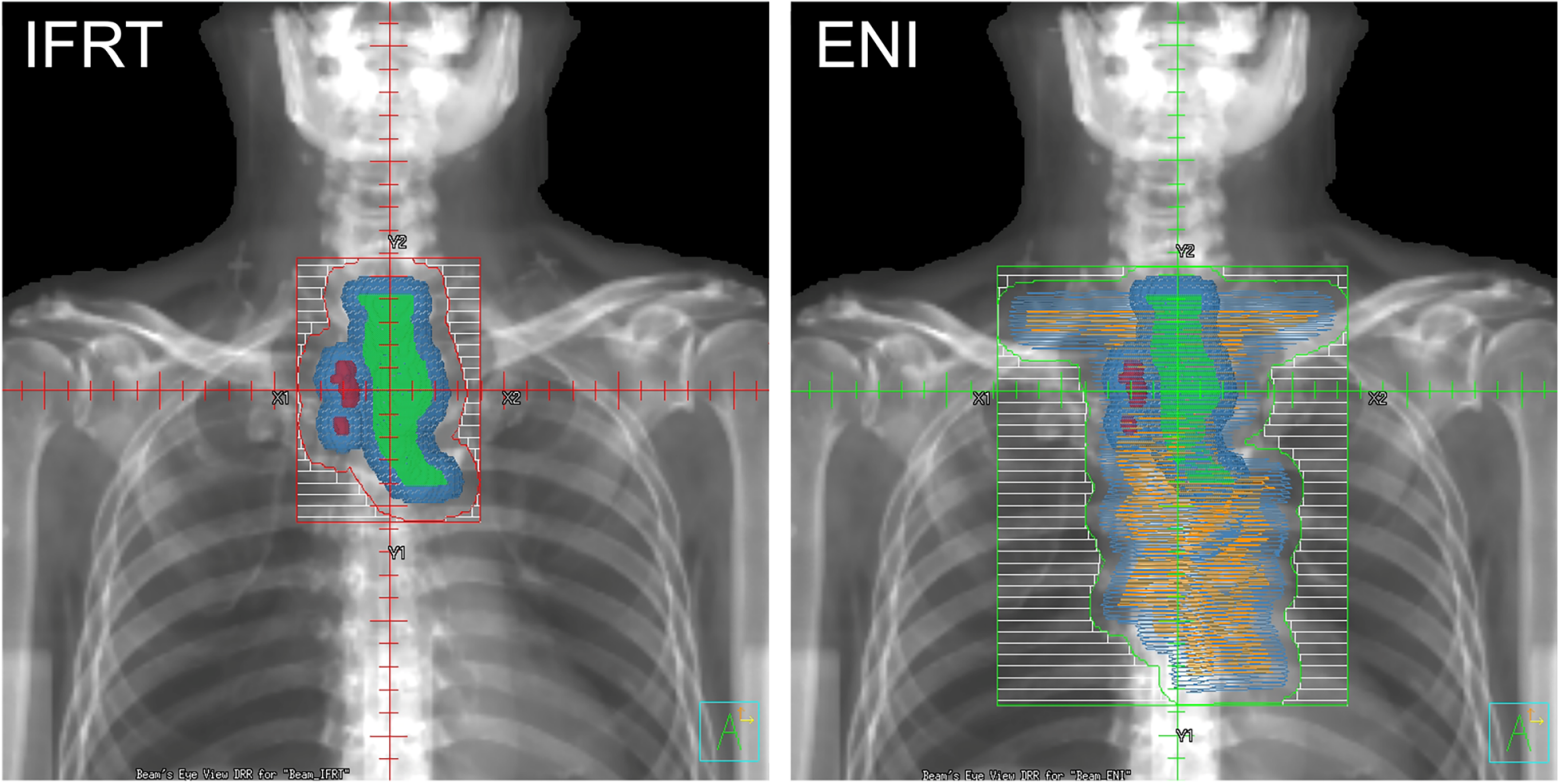
Irradiation fields for IFRT and ENI: CTVp (light green), CTVn (red), CTVprophy (orange), and PTV (light blue). CTVn, clinical target volume of the lymph nodes; CTVp, clinical target volume of the primary tumor; CTVprophy, clinical target volume of the prophylactic nodal area; ENI, elective nodal irradiation; IFRT, involved‐field radiotherapy; PTV, planning target volume.

A radiation oncologist delineated the gross tumor volume (GTV) on axial CT images using the Pinnacle treatment planning system (version 8.0m–9.10, Philips Medical Systems, Fitchburg, WI, USA), with reference to endoscopically placed clip markers by gastroenterologists, if applicable. The GTV was defined as any visible esophageal lesion (GTVp) or clinically involved node (GTVn). The GTVn criteria included (a) a tumor diameter of at least 1 cm in the short axis on CT, (b) a tendency for enlargement, or (c) positive fluorodeoxyglucose‐positron emission tomography (FDG‐PET) results before CRT. CTV was defined as CTVp, adding a 0–30‐mm margin along the esophagus from the GTVp in superior and inferior directions, as determined by individual radiation oncologists, and CTVn was equal to GTVn. An internal margin of 0–5 mm was added around the CTV to define the internal target volume (ITV) based on respiratory tumor motion measurements using radiographic fluoroscopy (SHIMADZU SAT‐20 [2012] and CANON LX‐40A [2013–2018]), with endoscopically placed clip markers as landmarks. The internal margin caused by respiratory motion was reduced by the breath‐hold technique using our original respiration self‐monitoring device called Abches (APEX Medical, Inc., Tokyo, Japan) or mask immobilization if the tumor motion exceeded 5 mm under free‐breathing.^9^ A setup margin of 5–10 mm was added around the ITV to define the PTV. The dose calculations were performed using a superposition algorithm. Figure 1 illustrates examples of the irradiation fields for IFRT and ENI.

### Chemotherapy

Patients received standard‐dose cisplatin/5‐fluorouracil (5‐FU) or low‐dose cisplatin/5‐FU‐based regimens based on the discretion of the attending physician. The former consisted of 70 mg/m^2^ cisplatin administered on days 1 and 29 combined with a continuous infusion of 700 mg/m^2^ 5‐FU administered on days 1–4 and 29–32. The latter comprised a 1‐h infusion of 5 mg/m^2^ cisplatin before RT combined with a continuous infusion of 250 mg/m^2^ 5‐FU on the first 5 days of each week. Chemotherapy was initiated on day 1 of irradiation. All patients underwent concurrent chemotherapy and RT. Neoadjuvant chemotherapy was permitted with the intention of preoperative treatment or in cases of tracheal invasion.

### Evaluation

The following clinical data were collected from the patient's medical records: age, sex, Eastern Cooperative Oncology Group performance status, weight change during RT, smoking history, drinking status, histology, clinical stage, primary tumor site, chemotherapy regimen, radiation dose, and respiratory motion management.

The initial tumor response was assessed using CT scans, following the Response Evaluation Criteria in Solid Tumors version 1.1, within 6 months of CRT completion.^10^

Disease failure was evaluated using posttreatment endoscopy, CT, or FDG‐PET and classified into three subgroups: (a) locoregional only, (b) locoregional with distant, and (c) distant only. Locoregional failure included esophageal and regional lymph node failure, classified as in‐field or out‐of‐field.

We also evaluated toxicities, primarily pneumonia, and esophagitis using the Common Terminology Criteria for Adverse Events (CTCAE) version 5.0. Furthermore, the incidence of esophageal fistulae was calculated in all patients after CRT.

### Statistical analysis

OS was defined as the time from RT initiation to death from any cause or last follow‐up. Progression‐free survival (PFS) was defined as the time from RT initiation to the first detection of disease progression, death from any cause, or last follow‐up. Locoregional failure‐free survival (LRFFS) was defined as the time from RT initiation to the first detection of locoregional failure, death from any cause, or last follow‐up. Locoregional control (LC) was defined as the time from RT initiation to the first detection of locoregional failure. The OS, PFS, LRFFS, and LC rates were calculated using the Kaplan–Meier method. Univariate analysis was performed using log‐rank tests. Logistic regression was used to estimate the odds ratios and 95% confidence intervals (CIs). Statistical significance was set at *p* < 0.05. All statistical analyses were performed using EZR (Saitama Medical Center, Jichi Medical University, Saitama, Japan), a graphical user interface for R (The R Foundation for Statistical Computing, Vienna, Austria), which is a modified version of the R commander designed to add statistical functions frequently used in biostatistics.^11^

## RESULTS

### Patient characteristics

Thirty‐five patients met the eligibility criteria. The baseline patient characteristics are presented in Table 1. Their median age was 71 years (range: 55–85 years). The median tumor length measured using CT was 5 cm (range, 2–14 cm). Eleven (31%) and 14 (40%) patients had T3 and T4 disease, respectively, and only supraclavicular lymph node metastases were included in the M1 staging.

**TABLE 1:** Patient characteristics.

Characteristics	*n*	%
Age (years)		
Range	55–85	NA
Median	71	NA
Sex		
Male	34	97
Female	1	3
Performance status		
0	21	60
1	12	34
2	2	6
Smoking history		
Never	4	11
Former	21	60
Current	10	29
Alcohol drinking		
Moderate	10	29
Heavy	25	71
Tumor main location		
Cervical	9	26
Upper thoracic	6	17
Middle thoracic	17	49
Lower thoracic	3	9
Tumor length (cm)		
Range	2–14	NA
Median	5	NA
Clinical stage		
I	5	14
II	5	14
III	11	31
IVA	9	26
IVB	5	14
T category		
T1	7	20
T2	4	11
T3	18	51
T4	6	17
N category		
N0	9	26
N1	4	11
N2	16	46
N3	6	17
M category		
M0	30	86
M1	5	14
FDG‐PET		
Yes	15	43
No	20	57
Bronchoscopy		
Yes	17	49
No	18	51

Abbreviations: FDG‐PET, fluorodeoxyglucose‐positron emission tomography; NA, not applicable.

### Treatment efficacy

Table 2 summarizes the patient treatment details. Notably, all patients completed RT, and 30 (86%) completed concurrent chemotherapy. Ten patients (29%) received neoadjuvant chemotherapy. Regarding respiratory motion management, 22 patients (63%) were treated with the breath‐hold technique, 10 (29%) with mask immobilization, and three (8%) with free breathing.

**TABLE 2:** Treatment characteristics.

Characteristics	*n*	%
RT modality		
3D‐CRT	30	86
IMRT	5	14
Total radiation dose		
66 Gy	21	60
70 Gy	14	40
Chemotherapy regimen		
Standard‐dose cisplatin/5‐FU	24	69
Low‐dose cisplatin/5‐FU	11	31
Neoadjuvant chemotherapy		
Yes	10	29
No	25	71
Respiratory motion management		
Breath‐hold	22	63
Mask immobilization	10	29
Free‐breathing	3	8

Abbreviations: 3D‐CRT, three‐dimensional conformal radiotherapy; 5‐FU, 5‐fluorouracil; IMRT, intensity‐modulated radiation therapy; RT, radiotherapy.

**FIGURE 2:**
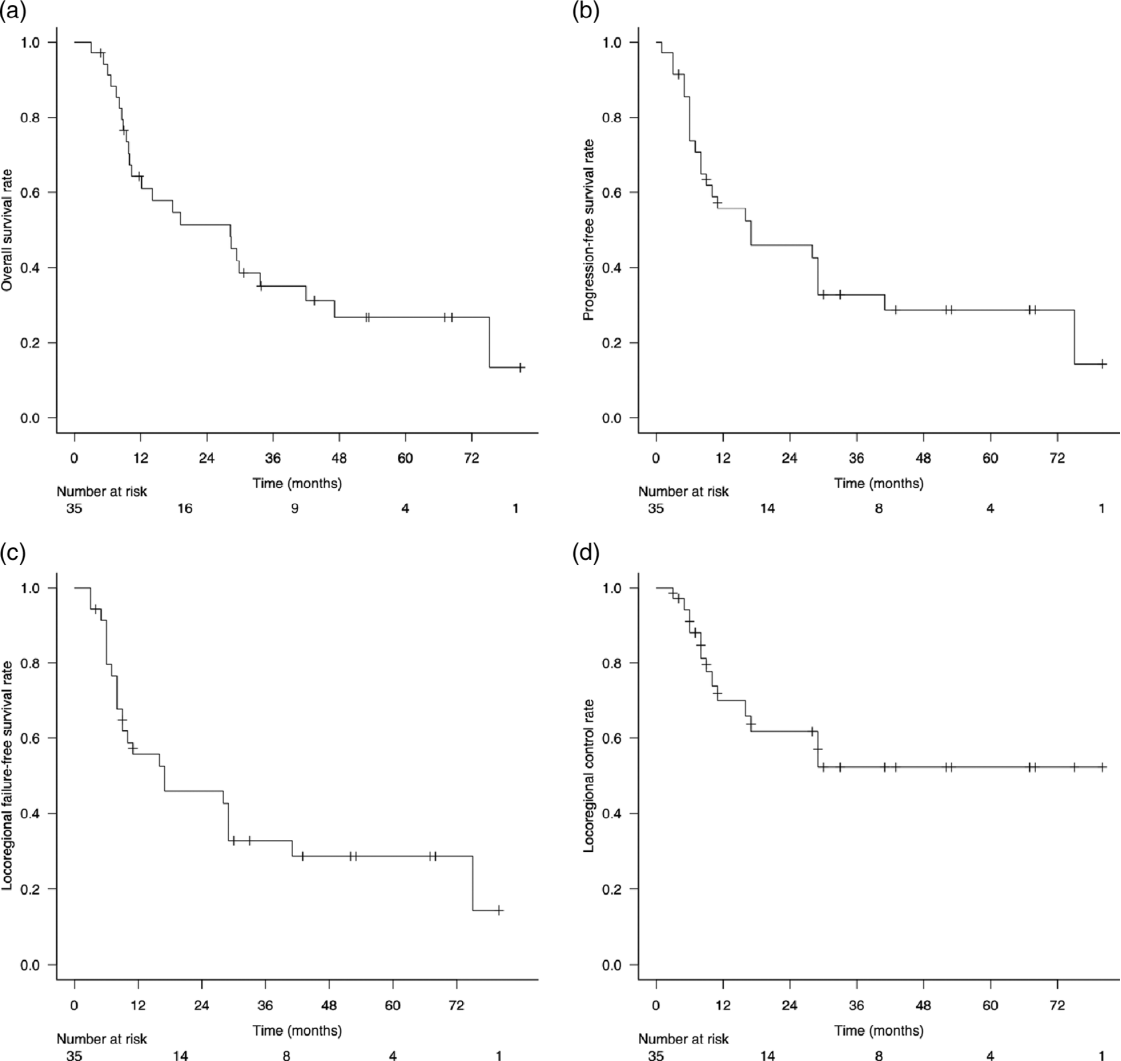
Survival curves of all cases: (a) OS, (b) PFS, (c) LRFFS, and (d) LC. LC, locoregional control; LRFFS, locoregional failure‐free survival; OS, overall survival; PFS, progression‐free survival.

**FIGURE 3:**
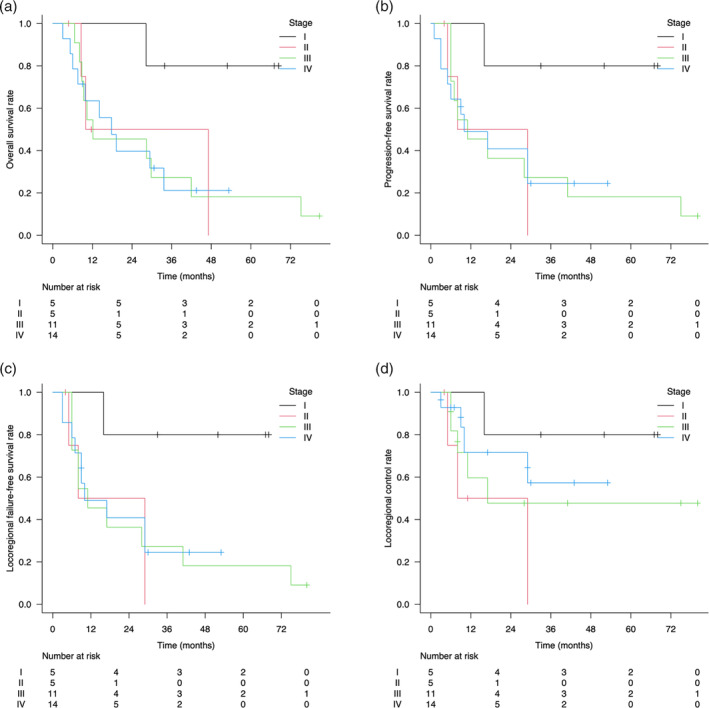
Survival curves of all cases according to the clinical stage: (a) OS, (b) PFS, (c) LRFFS, and (d) LC. LC, locoregional control; LRFFS, locoregional failure‐free survival; OS, overall survival; PFS, progression‐free survival.

**FIGURE 4:**
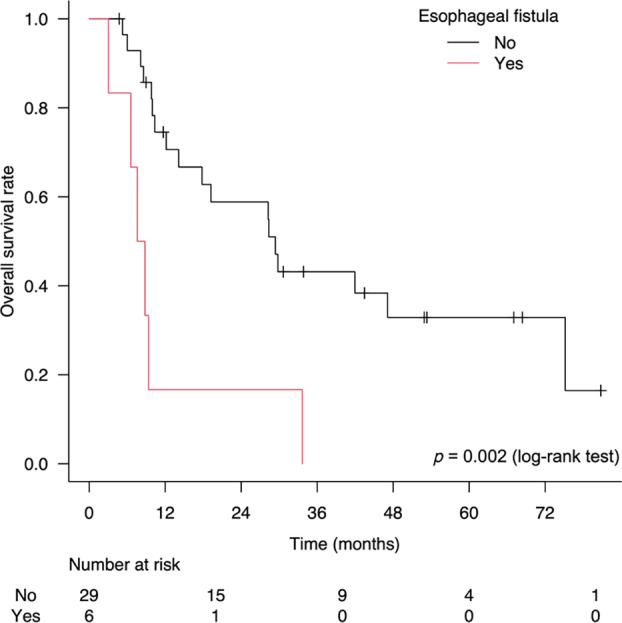
Overall survival rate according to esophageal fistula.

The median follow‐up times for survivors and all patients were 43 months (range, 4–80 months) and 17 months (range, 3–80 months), respectively. Thirty‐two of the 35 patients responded to treatment, leading to an overall response rate of 94.3%, with a complete response in 85.7% and a partial response in 8.6% of patients. The survival curves are shown in Figures 2 and 3. The 2‐year OS, PFS, LRFFS, and LC rates were 51.4% (95% CI, 33.3–66.9), 45.9% (95% CI, 28.4–61.7), 45.9% (95% CI, 28.4–61.7), and 61.7% (95% CI, 41.1–77.0), respectively. Exclusion of patients with stage IV disease resulted in a 2‐year OS of 59.6% (95% CI, 35.1–77.4). A significant difference in OS was observed between patients with and without esophageal fistulas after CRT (*p* = 0.002, log‐rank test) (Figure 4).

### Patterns of failure

The failure patterns are listed in Table 3. Sixteen patients (45.7%) experienced disease failure, including 10 (28.6%) with locoregional‐only failure, three (8.6%) with locoregional and distant failure, and three (8.6%) with distant‐only failure. Out‐of‐field nodal failure occurred in one patient (2.9%) with distant metastasis. Neoadjuvant chemotherapy was not administered in this case, and the lymph nodes that caused the out‐of‐field failure were small and showed no obvious FDG uptake before CRT.

**TABLE 3:** Patterns of failure.

	*n*	%
Total failure	16	45.7
Locoregional only	10	28.6
In‐field esophagus only	6	17.1
In‐field node only	2	5.7
In‐field esophagus with in‐field node	2	5.7
Locoregional with distant	3	8.6
In‐field esophagus with distant	2	5.7
Out‐of‐field node with distant	1	2.9
Distant only	3	8.6

### Toxicities

The toxicities during or after RT are listed in Table 4. Major grade 3 or higher toxicities included decreased white blood cell count (48.6%), neutrophil count (34.3%), and esophageal stenosis (31.4%); however, no grade 3 or higher cardiopulmonary toxicities were observed. Grade 5 toxicities included esophageal fistula in six patients (17.1%) and esophageal hemorrhage in two (5.7%). Among them, three cases of grade 5 esophageal fistula were defined as treatment‐related deaths, which showed a tumor response without locoregional failure.

**TABLE 4:** Summary of toxicities.

Toxicities	G1	G2	G3	G4	G5	≥G3
	*n*	*n*	*n*	*n*	*n*	*n* (%)
Dermatitis radiation	19	1	1	0	0	1 (28.5)
Esophagitis	7	20	5	0	0	5 (14.2)
Esophageal stenosis	2	6	11	0	0	11 (31.4)
Esophageal hemorrhage	0	0	2	0	2	4 (11.4)
Esophageal fistula	0	0	0	0	6	6 (17.1)
Pneumonia	23	4	0	0	0	0 (0)
Pleural effusion	8	6	0	0	0	0 (0)
Pericardial effusion	0	14	0	0	0	0 (0)
White blood cell count decrease	1	15	13	4	0	17 (48.6)
Neutrophil count decrease	1	15	7	5	0	12 (34.3)
Anemia	14	14	7	0	0	7 (20.0)
Platelet count decrease	23	6	3	2	0	5 (14.3)

Table 5 presents the details of cases of patients who developed esophageal fistula. The time‐to‐onset of esophageal fistula, defined as the interval between CRT completion and the date of fistula diagnosis, ranged from 1 to 22 months. The incidence rates of esophageal fistulas were 4.8% (66 Gy) and 35.7% (70 Gy). Table 6 shows the results of the univariate analysis of the risk factors for esophageal fistulas. Bronchial/tracheal compression by tumors and a higher RT dose (70 Gy) were significantly correlated with esophageal fistula incidences.

**TABLE 5:** Details of cases of patients who developed esophageal fistula.

Esophageal fistula	Age	Location	Clinical stage	Bronchial/tracheal compression	Total dose	Treatment response	Locoregional failure	Time to onset (months)	Clinical outcome
Case 1	65	Ut	T4bN2M1	Yes	70 Gy/35 fr	PR	No	1	Death from complications
Case 2	58	Mt	T3N3M0	Yes	70 Gy/35 fr	PR	No	2	Death from complications
Case 3	80	Ut	T3N1M0	Yes	70 Gy/35 fr	CR	No	6	Death from complications
Case 4	68	Mt	T3N2M0	Yes	70 Gy/35 fr	CR	Yes	6	Death from cancer
Case 5	65	Mt	T4bN2M0	Yes	70 Gy/35 fr	CR	Yes	22	Death from cancer
Case 6	85	Mt	T3N2M0	Yes	66 Gy/33 fr	SD	No	4	Death from cancer

Abbreviations: CR, complete response; Mt, middle thoracic; PR, partial response; SD, stable disease; Ut, upper thoracic.

**TABLE 6:** Results of univariate analysis of risk factors for esophageal fistula.

Variables	OR	95% CI	*p*‐value
Age			
<75	‐	‐	‐
≥75	0.823	0.064–6.917	1
Tumor length (cm)			
<5	‐	‐	‐
≥5	3.924	0.369–206.420	0.366
T category			
Non‐T4	‐	‐	‐
T4	2.999	0.207–31.538	0.268
N category			
N0–1	‐	‐	‐
N2–3	3.421	0.320–180.425	0.377
Bronchial/tracheal compression			
No	‐	‐	‐
Yes	Inf	2.016–Inf	0.003
Total radiation dose (Gy)			
<70	‐	‐	‐
70	10.328	0.962–549.079	0.028

Abbreviations: CI, confidence interval; OR, odds ratio.

## DISCUSSION

To our knowledge, this is the first study to evaluate the outcomes of high‐dose (≥66 Gy) IFRT combined with respiratory motion management in patients with ESCC. Table 7 summarizes the results of IFRT for ESCC using various doses and fractionation schedules. Our study differs from many others in that we focused on the internal and setup margins based on individual measurements of respiratory tumor motion. In some cases, the total margin exceeded 10 mm, particularly in the superior‐inferior direction, under free‐breathing conditions. Our respiratory motion data suggest that the conventional definition of a uniform CTV‐to‐PTV margin may be inaccurate and sometimes insufficient. Our study revealed acceptable OS and response rates despite 40% of the patients in the cohort having stage IV disease and a median age of >70 years. Furthermore, the total failure rates were relatively low (45.7%), including only 2.9% of the cases with out‐of‐field nodal failure. These data suggest that dose escalation using IFRT combined with respiratory motion management is a feasible approach for treating ESCC.

**TABLE 7:** Results of involved‐field radiotherapy for patients with esophageal cancer.

Author	Year	Clinical stage	*n*	Total radiation dose	Respiratory motion management	Internal margin	Setup margin	OS	LRFFS	Total failure	Out‐of‐field nodal failure
Yamashita^13^	2015	I–IV (IV: 29%)	119	50–50.4 Gy/25–28 fr	No	NA	5–10 mm	2 years: 58.7%	2 years: 61.0%	NA	1.7%
Kim^6^	2017	II–III	116	63 Gy/35 fr	No	NA	NA	NA	2 years: 69.1%	NA	8.6%
Zhu^14^	2021	II–IV (IV: 22%)	436	61.2 Gy/34 fr	No	NA	10 mm	3 years: 53.6%	NA	59.2%	8.5%
Li^8^	2022	II–IV (IV: 28%)	108	61.2 Gy/34 fr	No	NA	10 mm	2 years: 71.9%	2 years: 67.3%	47.2%	6.5%
Current study	2024	I–IV (IV: 40%)	35	66–70 Gy/33–35 fr	Yes	0–5 mm	5–10 mm	2 years: 51.4%	2 years: 45.9%	45.7%	2.9%

Abbreviations: LRFFS, locoregional failure–free survival; NA, not applicable; OS, overall survival.

The present study found no grade 3 or higher pulmonary or cardiac toxicities. Conversely, 11 (31.4%) patients developed grade 3 esophageal stenosis, and six (17.1%) developed grade 5 esophageal fistula. A recent retrospective cohort study identified that the incidence rate of esophageal fistulas was 30.1% among patients with clinically unresectable T4b ESCC treated with definitive CRT, and the risk was significantly higher in those with bronchial/tracheal invasion.^12^ In our study, the T category (T4 vs. non‐T4) was not significantly associated with the incidence of esophageal fistulas. However, bronchial/tracheal compression confirmed using CT before treatment was a risk factor for esophageal fistulas. This could be partly due to the underestimation of T staging, as bronchoscopy was not available for all cases in our study. Conversely, in some clinical situations where bronchoscopy might be omitted due to the rapid progression of ESCC, bronchial/tracheal compression on CT images could be an alternative risk factor for esophageal fistulas. Furthermore, our analysis suggests that esophageal fistulas are associated with shorter OS in patients with ESCC treated with CRT. These results indicated that a higher RT dose (70 Gy) is not recommended, particularly in patients with bronchial/tracheal compression.

This study has some limitations. First, this was a retrospective study from a single institution with a small sample size, including the use of various chemotherapy regimens, which may limit the generalizability of our results. Second, using FDG‐PET to define viable lymph node metastasis was not mandatory in our study, which may have influenced radiotherapy planning. However, the low incidence of out‐of‐field nodal failure indicates that the benefits of high‐dose IFRT outweigh this limitation. Third, it was partially impossible to distinguish whether severe toxicities, such as esophageal fistula, were associated with CRT or tumor progression. Fourth, it was difficult to compare the treatment results with those of previous reports because the patient characteristics varied in each report. Fifth, directly comparing the treatment efficacy between the 66 and 70 Gy groups was challenging because the prescription dose was determined at the physician's discretion, which resulted in both groups not being equivalent in terms of patients' characteristics, such as age and clinical stage. Finally, treatment‐related toxicities may have been underestimated because of the study's retrospective nature.

In conclusion, we presented the treatment results of high‐dose (≥66 Gy) IFRT with respiratory motion management. This approach may be a feasible treatment option for ESCC regarding local control. However, a comprehensive assessment of the esophageal fistula risk based on clinical features is required to identify suitable candidates for high‐dose IFRT, which is not recommended, particularly in patients with bronchial/tracheal compression.

## AUTHOR CONTRIBUTIONS

All authors had full access to the data in the study and take responsibility for the integrity of the data and the accuracy of the data analysis. *Conceptualization*: M.M. *Supervision*: T.K. and H.O. *Writing—original draft*: M.M. *Writing—review and editing*: T.K., K.M., and H.O. *Investigation*: T.K., K.M., S.A., and T.A. *Resources*: N.S. and H.S. *Data curation*: M.S. and H.N.

## CONFLICT OF INTEREST STATEMENT

The authors declare no conflicts of interest.

## PATIENT CONSENT STATEMENT

Informed consent was obtained using an opt‐out method. Participants were provided with information about the study and were given the opportunity to decline participation. Those who did not opt‐out were considered to have given their consent.

## Data Availability

The data that support the findings of this study are available from the corresponding author, Masaki Matsuda, upon reasonable request.

## References

[1] Sung H , Ferlay J , Siegel RL , et al. Global cancer statistics 2020: GLOBOCAN estimates of incidence and mortality worldwide for 36 cancers in 185 countries. CA Cancer J Clin. 2021;71:209–249. 33538338 10.3322/caac.21660

[2] National Cancer Registry (Ministry of Health, Labour and Welfare), Tabulated by Cancer Information Service, National Cancer Center, Japan . Cancer statistics in Japan. https://ganjoho.jp/reg_stat/statistics/data/dl/en.html. Accessed 6 Jun 2024.

[3] Watanabe M , Otake R , Kozuki R , et al. Recent progress in multidisciplinary treatment for patients with esophageal cancer. Surg Today. 2020;50:12–20. 31535225 10.1007/s00595-019-01878-7 PMC6952324

[4] Cooper JS , Guo MD , Herskovic A , et al. Chemoradiotherapy of locally advanced esophageal cancer: long‐term follow‐up of a prospective randomized trial (RTOG 85‐01). Radiation Therapy Oncology Group. JAMA. 1999;281:1623–1627. 10235156 10.1001/jama.281.17.1623

[5] Minsky BD , Pajak TF , Ginsberg RJ , et al. Int 0123 (Radiation Therapy Oncology Group 94‐05) phase III trial of combined‐modality therapy for esophageal cancer: high‐dose versus standard‐dose radiation therapy. J Clin Oncol. 2002;20:1167–1174. 11870157 10.1200/JCO.2002.20.5.1167

[6] Kim HJ , Suh YG , Lee YC , et al. Dose‐response relationship between radiation dose and loco‐regional control in patients with stage II–III esophageal cancer treated with definitive chemoradiotherapy. Cancer Res Treat. 2017;49:669–677. 27737537 10.4143/crt.2016.354 PMC5512369

[7] Nayan N , Bhattacharyya M , Jagtap VK , Kalita AK , Sunku R , Roy PS . Standard‐dose versus high‐dose radiotherapy with concurrent chemotherapy in esophageal cancer: a prospective randomized study. South Asian J Cancer. 2018;7:27–30. 29600230 10.4103/sajc.sajc_178_17 PMC5865091

[8] Li H , Fang Y , Gu D , et al. Paclitaxel and cisplatin combined with concurrent involved‐field irradiation in definitive chemoradiotherapy for locally advanced esophageal squamous cell carcinoma: a phase II clinical trial. Radiat Oncol. 2022;17:105. 35681233 10.1186/s13014-022-02078-3 PMC9185874

[9] Onishi H , Kawakami H , Marino K , et al. A simple respiratory indicator for irradiation during voluntary breath holding: a one‐touch device without electronic materials. Radiology. 2010;255:917–923. 20501729 10.1148/radiol.10090890

[10] Eisenhauer EA , Therasse P , Bogaerts J , et al. New response evaluation criteria in solid tumours: revised RECIST guideline (version 1.1). Eur J Cancer. 2009;45:228–247. 19097774 10.1016/j.ejca.2008.10.026

[11] Kanda Y . Investigation of the freely available easy‐to‐use software ‘EZR’ for medical statistics. Bone Marrow Transplant. 2013;48:452–458. 23208313 10.1038/bmt.2012.244 PMC3590441

[12] Chen B , Deng M , Yang C , et al. High incidence of esophageal fistula on patients with clinical T4b esophageal squamous cell carcinoma who received chemoradiotherapy: A retrospective analysis. Radiother Oncol. 2021;158:191–199. 33667583 10.1016/j.radonc.2021.02.031

[13] Yamashita H , Takenaka R , Omori M , et al. Involved‐field radiotherapy (IFRT) versus elective nodal irradiation (ENI) in combination with concurrent chemotherapy for 239 esophageal cancers: a single institutional retrospective study. Radiat Oncol. 2015;10:171. 26269033 10.1186/s13014-015-0482-9 PMC4554303

[14] Zhu H , Rivin del Campo E , Ye J , et al. Involved‐field irradiation in definitive chemoradiotherapy for locoregional esophageal squamous cell carcinoma: results from the ESO‐Shanghai 1 trial. Int J Radiat Oncol Biol Phys. 2021;110:1396–1406. 33677048 10.1016/j.ijrobp.2021.02.053

